# Supplementary material for: Meniscal tear outcome Study (METRO Study): a study protocol for a multicentre prospective cohort study exploring the factors which affect outcomes in patients with a meniscal tear
Source: BMJ Open. 2020 Jul 12;10(7):e038681. doi: 10.1136/bmjopen-2020-038681 (PMC7359070; doi:10.1136/bmjopen-2020-038681)
Supplement: Author's manuscript [file bmjopen-2020-038681.draft_revisions.pdf]

BMJ Open is committed to open peer review. As part of this commitment we make the peer review history of every article we publish publicly available.

When an article is published we post the peer reviewers' comments and the authors' responses online. We also post the versions of the paper that were used during peer review. These are the versions that the peer review comments apply to.

The versions of the paper that follow are the versions that were submitted during the peer review process. They are not the versions of record or the final published versions. They should not be cited or distributed as the published version of this manuscript.

BMJ Open is an open access journal and the full, final, typeset and author-corrected version of record of the manuscript is available on our site with no access controls, subscription charges or pay-per-view fees (http://bmjopen.bmj.com).

If you have any questions on BMJ Open's open peer review process please email info.bmjopen@bmj.com

BMJ Open

The Meniscal tear outcome study (METRO Study): A study protocol for a multicentre prospective cohort study.

Journal:	*BMJ Open*
Manuscript ID	bmjopen-2020-038681
Article Type:	Protocol
Date Submitted by the Author:	19-Mar-2020
Complete List of Authors:	Ahmed, Imran; University of Warwick, Clinical Trials Unit<br>Bowes, Mike; IMorphics Limited<br>Hutchinson, Charles; University of Warwick, Warwick Medical School<br>Parsons, Nicholas; University of Warwick, Warwick Medical School<br>Staniszewska, Sophie; University of Warwick, Warwick Medical School<br>Price, Andrew; University of Oxford, Nuffield Department of Orthopaedics, Rheumatology and Musculoskeletal Sciences<br>Metcalfe, Andrew; University of Warwick Warwick Medical School, Warwick Clinical Trials Unit; University Hospitals Coventry and Warwickshire NHS Trust, Trauma and Orthopaedics
Keywords:	Adult orthopaedics < ORTHOPAEDIC & TRAUMA SURGERY, Knee < ORTHOPAEDIC & TRAUMA SURGERY, ORTHOPAEDIC & TRAUMA SURGERY

SCHOLARONE™
Manuscripts

I, the Submitting Author has the right to grant and does grant on behalf of all authors of the Work (as defined in the below author licence), an exclusive licence and/or a non-exclusive licence for contributions from authors who are: i) UK Crown employees; ii) where BMJ has agreed a CC-BY licence shall apply, and/or iii) in accordance with the terms applicable for US Federal Government officers or employees acting as part of their official duties; on a worldwide, perpetual, irrevocable, royalty-free basis to BMJ Publishing Group Ltd ("BMJ") its licensees and where the relevant Journal is co-owned by BMJ to the co-owners of the Journal, to publish the Work in this journal and any other BMJ products and to exploit all rights, as set out in our licence.

The Submitting Author accepts and understands that any supply made under these terms is made by BMJ to the Submitting Author unless you are acting as an employee on behalf of your employer or a postgraduate student of an affiliated institution which is paying any applicable article publishing charge ("APC") for Open Access articles. Where the Submitting Author wishes to make the Work available on an Open Access basis (and intends to pay the relevant APC), the terms of reuse of such Open Access shall be governed by a Creative Commons licence – details of these licences and which Creative Commons licence will apply to this Work are set out in our licence referred to above.

Other than as permitted in any relevant BMJ Author's Self Archiving Policies, I confirm this Work has not been accepted for publication elsewhere, is not being considered for publication elsewhere and does not duplicate material already published. I confirm all authors consent to publication of this Work and authorise the granting of this licence.

For peer review only

The Meniscal tear outcome study (METRO Study): A study protocol for a multicentre prospective cohort study.

Imran Ahmed (Imran.ahmed4@nhs.net)

Mike Bowes (mike@imorphics.com)

Charles Hutchinson (c.e.hutchinson@warwick.ac.uk)

Nick Parsons (nick.parsons@warwick.ac.uk)

Sophie Staniszewska (sophie.staniszewska@warwick.ac.uk)

Andrew Price (Andrew.price@ndorms.ox.ac.uk)

Andrew Metcalfe (a.metcalfe@warwick.ac.uk)

Corresponding author:

Imran Ahmed

NIHR Doctoral Research Fellow

Warwick Clinical trials unit

Clinical sciences and research laboratories

University Hospital Coventry and Warwickshire

Clifford Bridge Road

Coventry

CV2 2DX

Imran.ahmed4@nhs.net

Word Count:

For peer review only

Abstract

Introduction

This study is designed to explore the baseline characteristics of patients under 55 years of age with a meniscal tear, and to describe the relationship between the baseline characteristics and patient reported outcome measures over 12 months. Research has highlighted the need for a trial to investigate the effectiveness of arthroscopic meniscectomy in younger patients. Before this trial, we need to understand the patient population in greater detail.

Methods and analysis

This is a multicentre prospective cohort study. Participants aged between 18 to 55 years with an MRI confirmed meniscal tear are eligible for inclusion. Baseline characteristics including age, body mass index, gender, participant-reported outcome measures (PROMs) duration of symptoms and magnetic resonance imaging (MRI) will be collected. The primary outcome measure is the Western Ontario Meniscal Evaluation Tool (WOMET) at 12 months. Secondary outcome measures will include PROMs such as EQ5D, Knee injury and osteoarthritis outcome score (KOOS) and patient global impression of change score at 3, 6 and 12 months.

Ethics and dissemination

The study obtained approval from the National Research Ethics Committee (NRES) West Midlands – Black Country research ethics committee (19/WM/0079) on 12th April 2019. The study is sponsored by the University of Warwick. The results will be disseminated via peer reviewed publication.

Registration details

UHCW R&D Reference: IA428119.

University of Warwick Sponsor ID: SC.08/18-19

Article summary

Strengths and limitations

- Multicentre prospective design
- Clearly defined outcome measures
- Comprehensive clinical and radiological assessment of participants
- Absence of clinical outcome measures

For peer review only

Introduction

Meniscal tears affect 60-70 per 100,000 of the population and account for 70,000 hospital admissions per year (1) (2). Over recent years, there has been a substantial increase in meniscus related literature, with a number studies questioning the effectiveness of surgical management(3-5).

The meniscus is a highly specialised c-shaped structure located within the knee (6). It has an important role in distributing load across the knee joint, preventing damage to the articular cartilage (7). Biomechanical studies have demonstrated the menisci distribute 70% of the load across the knee (8). In addition to load distribution, the menisci are involved in the stabilisation of the knee joint (7).

The meniscus is susceptible to tears or damage. Meniscal tears typically result from high energy twisting injuries, such injuries often occur during sporting activities (9, 10). Meniscal pathology is also common in older people following low energy trauma in the context of degeneration within the knee joint. Meniscal disease is closely associated with osteoarthritis (OA) and age-relation degeneration which can lead to changes in the histology or the menisci and their mechanical properties(11).

The current management of meniscal tears includes non-surgical options such as provision of advice and observation, physiotherapy or pharmacological options such as anti-inflammatory medication or intra-articular corticosteroid injections. Surgical options include meniscal repair (in limited cases) or arthroscopic partial meniscectomy (APM), a keyhole procedure performed with the intention of removing the torn or unstable meniscus which may be thought to cause pain or mechanical symptoms e.g. locking (12).

Previous randomised controlled trials have found no evidence for a difference in outcome between APM and physiotherapy in patients with a meniscal tear and co-existing arthritis (13). This lead to a change in NICE guidelines advising against arthroscopic surgery to treat meniscal tears in the context of established arthritis (18). Abram et al performed a systematic review including studies comparing arthroscopic meniscectomy versus a comparator in patients with a meniscal tear(4). The authors included 10 studies for analysis and performed subgroup analysis for patients without OA. The authors reported a small improvement in knee pain for all patients with a meniscal tear in the meniscectomy group compared to the non-surgical group (standardised mean difference (SMD) 0.22 95% CI 0.04 to 0.4). Three studies reported pain scores in patients without OA, in which the authors found an increased improvement in pain scores in the meniscectomy group (SMD 0.35 95% CI 0.04 to 0.66). There was a similar improvement in knee function in the meniscectomy group in patients without OA compared to all patients undergoing meniscectomy (SMD 0.18 vs SMD 0.3).

Despite the evidence challenging the clinical effectiveness of APM, there has been an overall increase in the number of APMs performed over the last 20 years (14-16). Previous literature has provided evidence on the lack of effectiveness of APM in patients with a degenerative knee or arthritis as the symptoms may be caused by the degeneration and not the meniscal tear. International consensus from specialist knee societies has highlighted the importance of patient factors which are important in the management of meniscal tears. For example, patients with a particular pattern of meniscal tear visible on MRI ('a meniscal target') and the corresponding pattern of symptoms or signs may benefit from APM. Furthermore, patients should undergo a period of non-operative management before referral for APM (17).

Previous evidence supports the view that APM may not be beneficial in patients with meniscal tears and degeneration or arthritis within the knee joint (18). Given the heterogeneous nature of the

For peer review only

condition, it may be that APM is effective in patients without these signs of degeneration. There is a view amongst researchers and clinicians that surgery may be beneficial amongst a particular subset of patients and further work is needed to identify this subset (4). A Danish cohort study was performed in order to identify features which may predict variability in patient reported outcomes (PROMs) for patients following arthroscopy. The authors included 18 preoperative factors and found the strongest predictive factors were no previous meniscal surgery and more severe preoperative knee symptoms. The models overall predictive performance was very low (optimum adjusted R^2=0.080) and showed poor calibration (calibration slope 0.772) (5). The authors only included patients undergoing operative intervention, work is needed to identify outcomes in all patients that are managed with a meniscal tear, in order to identify which patients performed well with non-operative management and which perform poorly. This could explain why the literature reports that up to 30% of patients cross over into the surgery group (4). Another weakness of this work is that the authors did not include MRI based features into the model. The British Association for Surgery of the Knee (BASK) guidelines suggest that radiology investigations should be included in treatment decisions, with certain patterns of tears being ‘target’ lesions. A future cohort study will need to assess the importance of the MRI tear pattern as an outcome predictor.

A well-designed cohort study needs to be performed with patients being recruited at the point of diagnosis or presentation to an orthopaedic specialist. This will allow researchers to include patients being managed non-operatively. By including MRI analysis in a potential predictive model, this will produce a comprehensive model to identify which features can predict variability in PROMs.

Study Aim

To describe the baseline characteristics and imaging findings of young patients with a meniscal tear and to explore the relationship, if any, between these baseline features and 12-month outcomes.

We will address the following research objectives:

1. Describe the baseline characteristics, pattern of symptoms and imaging findings of younger people (aged < 55 years) with a meniscal tear (study population)

2. Explore the relationship between these features and 12-month PROMS.

Methods and Analysis

A multi-centre prospective cohort study will be performed. The study obtained approval from the National Research Ethics Committee (NRES) West Midlands – Black Country research ethics committee (19/WM/0079) on 12th April 2019. All patients presenting to a secondary care centre (elective knee clinic or acute knee clinic) with a magnetic resonance imaging (MRI) confirmed meniscal tear, under the care of orthopaedic consultants at any of the participating sites, will be invited to take part in the study. The following eligibility will be implemented for patient selection.

Inclusion criteria:

- Age between 18 and 55 years
- Presence of an MRI confirmed meniscal tear
- Provision of informed written consent

Exclusion criteria:

For peer review only

- Anterior cruciate ligament or other major knee ligament injury. This does not include a previous unrelated healed medial collateral ligament (MCL) tear or a meniscal root tear (which is considered a type of meniscal tear in this study)
- Associated intra-articular fracture of the tibial plateau or femur. Previous fractures not thought to be related to the tear are not an exclusion criteria for the study
- Previous knee surgery, e.g unicondylar knee replacement; total knee replacement; knee arthroscopy; meniscal repair or meniscectomy.
- Previous entry into the present study (i.e. the other knee)
- Participant is unable to undertake study procedures

Participant identification and screening

Potential participants will be identified by a member of the attending clinical team or a member of the clinical research team by screening clinic lists in secondary or intermediate care clinics. This will involve a member of the clinical team reviewing letters for the upcoming knee or orthopaedic clinics to identify potential participants.

Eligibility will be confirmed based on assessment and standard care MRI. In normal clinical practice, MRI is a requirement for diagnosis of a meniscal tear, therefore all participants entering the study will already have had one at the time of entry into the study.

All individuals who meet the study entry criteria will be checked for eligibility and recorded on the monthly screening log. Eligible potential participants who are willing to be approached by a suitably trained member of the research team will be provided with verbal and written information about the study, and will have the opportunity to discuss and ask questions.

Baseline data collection

Once participants have provided informed consent, baseline data will be collected by the research team. Baseline characteristics such as age, gender, body mass index and date of potential injury and duration of symptoms will be collected. Baseline PROMs will be collected at recruitment in order to assess changes in PROMs at 12 months. Clinical features which are consistent with a treatable meniscal lesion, outlined by the BASK consensus meeting, will also be collected from the attending clinician (for a locked knee) and the participant (for locking and catching (17). These include the presence of:

- Locked knee: sudden onset, complete mechanical block to flexion or extension of the knee, detected on clinical examination and which does not resolve despite adequate analgesia.
- Locking: an intermittent block to normal range of movement of the knee (commonly a block to extension) with an associated unlocking movement. Knee returns to near normal after unlocking.
- Catching: the sensation of something intermittently out of place in the knee and interfering with joint movement.

These will be assessed on initial baseline questionnaires which will be provided by the host site. If the clinician documents specific examination findings (in particular alignment), this will be collected by the study team.

MRI scans will be analysed to assess:

1. Anatomical location and tear pattern. Tear patterns include the following which have been recognised by BASK as potentially treatable ‘meniscal targets’(17).

For peer review only

a) *Bucket handle tear: a longitudinal tear involving more than 25% of the meniscus length (can be displaced or undisplaced)*
b) *Displaced meniscal tear: fragments are displaced from their usual anatomical position*
c) *Meniscal root tear*
d) *Radial tear: a vertical tear which may or may not extend into the meniscocapsular junction*
e) *Horizontal tear*
f) *Complex tear: a meniscal lesion with more than one place of tear in continuity*

2. Whole-Organ Magnetic Resonance Imaging Score (WORMS): This is a semi-quantitative, multi-feature scoring method for whole-organ evaluation of the knee using conventional MRI. It is based on 14 features include cartilage and meniscal integrity (19).

3. Bone area: analysis of bone area and cartilage volume using proprietary semi-automated segmentation software in collaboration with IMorphics (IMorphics Limited, Stryker House, Hambridge road, Newbury, Berkshire). Previous research has demonstrated that change in bone area is a more sensitive marker of osteoarthritis than cartilage thickness or joint space narrowing. This change in bone area could lead to the development of meniscal tears as flattening of the femoral condyls could potentially reduce the space available for the meniscus increasing the likelihood of meniscal damage (20).

PROMs will be collected at recruitment, as a baseline for PROMS at 12 months (primary outcome). With follow up will be at three months, six months and 12 months. These data will be collected by a number of methods (either postal, telephone, electronically or face- to- face,) depending on participant preference. If a participant requests face-to-face consultation or has difficulty reading or writing, a clinic visit will be arranged

Primary outcome:

Western Ontario Meniscal Evaluation Tool (WOMET) at 12 months. This is a meniscal tear disease-specific quality of life measure developed in collaboration with patients in 2007. It consists of 16 questions (items) focussing on three domains: (i) physical symptoms, (ii) sport/recreation/work/lifestyle and (iii) emotions. Items are assessed on a visual analogue scale (VAS), summed and reported as a percentage of the maximum score of 1600 (21). A recent systematic review of PROMS used for meniscal research demonstrated that WOMET had the strongest evidence for content validity (22). It is also one of the only PROMS developed with the involvement of patients with meniscal tears as their primary diagnosis.

Secondary outcomes

- **WOMET:** collected at baseline, 3 and 6 months: as outlined above.
- **Knee injury and Osteoarthritis outcome score 4 (KOOS)(26):** The KOOS questionnaire was developed in 1998 as a knee injury specific outcome measure of patients at risk of developing arthritis(23). Domains are pain, symptoms, functional status, sports activity and quality of life. It is used extensively in knee injury research, and has been the primary outcome in the majority of clinical trials studying the treatment of meniscal tears. In previous meniscal tear research a shorter form of KOOS ($KOOS_4$) has been widely used (24). This focuses on four out of five of the domains pain, symptoms, sport and recreational function and quality of life. Standardised answer options are provided using a Likert scale and each question is assigned a score of 0 – 4. A score of 100 indicates no symptoms whereas a score of 0 indicates extreme symptoms. This outcome tool can be used as a postal

For peer review only

or electronic survey and both versions are comparable with regards to psychometrics (25). KOOS4 will be collected at baseline and 12 months only.

- **EuroQol (EQ-5D-5L):** This is a validated measure of health-related quality of life, consisting of a five-dimension health status classification system and a separate visual analogue scale. EQ-5D is applicable to a wide range of health conditions and treatments and provides a simple descriptive profile and a single index value for health status, range from -0.594 to 1, and anchored at zero (death) (26, 27). EQ-5D is primarily designed for self-completion by respondents and is ideally suited for use in postal surveys, in clinics and face-to-face interviews (28, 29). It is cognitively simple, taking only a few minutes to complete (27, 30). This will be collected at baseline, 3 months, 6 months and 12 months.
- **Patient global assessment of change (PGIC):** A simple 7 point scale assessing participant perception of improvement (31). A standardised question will be used to explore the change in activity limitations, symptoms and quality of life in the painful knee. Responses will range from 'no change or condition has worsened' to 'a great deal better. This will provide valuable information for a future large scale trial by providing information on the minimally clinical important difference in WOMET score for this patient population. This will be collected at 3 months, 6 months and 12 months.
- **Surgery:** We will also collect data on whether participants undergo surgery for their meniscal tear. At each time point (3, 6 and 12 months) patients will be asked if they underwent surgery. We will continue to follow up patients after surgery to determine their outcome in a pragmatic fashion, as would happen in the conservative arm of a large trial.

Sample size calculation

Previous experience (32) for studies of this type, where statistical models have been built to explain variation in PROMs, suggests that they rarely explain more than 10-20% of the variation in outcomes (R^2) at 12 months. Using a large (and therefore rather conservative) estimate for the numerator number of degrees of freedom of 25 (i.e. the model complexity) for a putative F test of the model significance and an $R^2 = 0.1$, suggests a sample size of 160 for 90% power at the 5% significance level (https://cran.r-project.org/web/packages/pwr/; pwr.f2.test) (33). Experience of previous such studies in similar settings have reported follow up rates of 80%. Assuming loss to follow up of 20%, 200 participants will be required for this study.

Statistical analysis

Summary statistics will be used to describe baseline data. We will report means and standard deviations (95% confidence intervals) for approximately normally distributed data and for other cases report the median and interquartile range. Mean 12 month PROMs scores will be reported for the whole cohort. We will also report mean 12 month PROMs for both the non-operative and operative group. Pearson correlation coefficients will be used to assess evidence for associations between baseline features and 12 month PROMs scores. A model will be developed relating the response variable (12-month PROM) to a number of explanatory variables (e.g. data collected at baseline). The type and structure of the models used will depend on the distribution of the response variable, and the nature of the association between the response and explanatory variable. A Linear model will be the initial choice in order to predict the response variable (12-month PROM) as a linear weighted sum of the explanatory variables. The 'weights' or parameters of this model are estimated using regression analysis; providing us with regression coefficients (parameter estimates), that characterise the association.

For peer review only

A longitudinal anchor based method will be used to calculate the MCID. We will report the mean WOMET score for the participants in the 'almost the same, hardly any change' cohort and the mean WOMET score for the participants in the 'moderately better, and slight but noticeable change' cohort. The difference in the mean WOMET score will represent the MCID. This methodology has been described previously (34).

Model fitting and analysis will be undertaken in R (R Core team (2013) R Foundation for statistical computing, Vienna, Austria) (35).

Patient and public involvement

Eighteen patients with knee pain or osteoarthritis were consulted prior to study set up. They provided valuable input on study set up such as sources of recruitment, completion of case report forms, completion of questionnaires and how to conduct follow up. These patients also reviewed the language used in patient information sheets and consent forms to ensure it was appropriate for the target population.

We are in the process of setting up a PPI reference group of five patients with a meniscal tear. They will help address any issues which arise during the study.

Patient and public members will be involved in the interpretation of the results and facilitate the dissemination of the study results by helping to prepare patient leaflets, manuscripts for peer reviewed publication and dissemination through social media.

Patient and public members will also be regularly updated on study progress and will provide advice on issues including recruitment.

PPI will be reported using GRIPP2 reporting checklist(36).

Ethics and dissemination

The study obtained approval from the National Research Ethics Committee (NRES) West Midlands – Black Country research ethics committee (19/WM/0079) on 12th April 2019.

We will aim to publish the results in a peer reviewed journal, within 12 months of the study completing (Recruitment began in April 2019 and will continue to November 2020). The results of the study will also be disseminated via patient information material produced in collaboration with the PPI group. All key study findings will be presented at national and international conferences, e.g. British Orthopaedic Association (BOA) or British Association of Specialist Knee surgeons (BASK), and the American Academy of Orthopaedic Surgeons (AAOS).

Acknowledgements

We would like to acknowledge the support of:

1. The University of Warwick (Lead Sponsor)
2. University Hospital Coventry and Warwickshire (Lead NHS site).
3. Members of the PPI group.
4. Research and Clinical staff at all recruiting site: University Hospital Coventry and Warwickshire, George Elliot Hospital NHS Trust, University Hospitals Birmingham NHS Trust, Royal Orthoapedic Hospital, Birmingham Community Healthcare NHS trust, Robert Jones and Agnes Hunt NHS trust, Oxford University Hospitals, Imperial College NHS trust and North Bristol NHS trust.

For peer review only

For peer review only

Author contributions

IA: Study conception, study design, drafted and reviewed final manuscript.

MB: Study design, drafted and approved final manuscript.

CEH: Study design, drafted and approved final manuscript.

NP: Study design, drafted and approved final manuscript.

SS: Study design, drafted and approved final manuscript.

AP: Study design, drafted and approved final manuscript.

AM: Chief Investigator, Study design, drafted and approved final manuscript.

Funding statement

The study protocol represents research funded by a National Institute for Health Research (NIHR) Doctoral Fellowship Award (DRF-2018-11-ST2-030). The study is sponsored by the University of Warwick. The study sponsor provides ultimate approval of all new versions of the protocol before they become live. Both the funder and sponsor are required to provide final approval before publication of any study material.

Data management and sharing

All data collected will be anonymised after the collection of the baseline demographic data for each participant. Confidentiality will be strictly maintained and names or addresses will not be disclosed to anyone other than the staff involved in running the study. Participants will be identified by ID number initials. Identifiable participant data will be held in a locked filing cabinet and coded with the study number to tag identifiable data to the outcome data.

Direct access to source data/documents will be available for study-related monitoring or audit by Warwick CTU for internal audit, regulatory authorities or ethics committees.

Data will be available for non-commercial companies upon reasonable request**.**

Disclaimer

The study funder and sponsor had no role in the study design; the collection, analysis, or interpretation of data; the writing of the report; or the decision to submit for publication. The researchers are independent and the views expressed are those of the authors and not necessarily those of the NHS, the NIHR or the Department of Health.

Competing interests statement

Mike Bowes is a senior director of clinical applications at IMorphics limited.

References

1. Logerstedt DS, Snyder-Mackler L, Ritter RC, Axe MJ. Knee pain and mobility impairments: meniscal and articular cartilage lesions. The Journal of orthopaedic and sports physical therapy. 2010;40(6):A1-a35.
2. Abram SGF, Judge A, Beard DJ, Price AJ. Adverse outcomes after arthroscopic partial meniscectomy: a study of 700 000 procedures in the national Hospital Episode Statistics database for England. Lancet. 2018;392(10160):2194-202.

3. Abram SGF, Beard DJ, Price AJ. Arthroscopic meniscal surgery: a national society treatment guideline and consensus statement. Bone Joint J. 2019;101-B(6):652-9.
4. Abram SGF, Hopewell S, Monk AP, Bayliss LE, Beard DJ, Price AJ. Arthroscopic partial meniscectomy for meniscal tears of the knee: a systematic review and meta-analysis. Br J Sports Med. 2019.
5. Pihl K, Ensor J, Peat G, Englund M, Lohmander S, Jorgensen U, et al. Wild goose chase - no predictable patient subgroups benefit from meniscal surgery: patient-reported outcomes of 641 patients 1 year after surgery. Br J Sports Med. 2019.
6. Smith NA, Costa ML, Spalding T. Meniscal allograft transplantation: rationale for treatment. The bone & joint journal. 2015;97-b(5):590-4.
7. Masouros SD, McDermott ID, Amis AA, Bull AM. Biomechanics of the meniscus-meniscal ligament construct of the knee. Knee surgery, sports traumatology, arthroscopy : official journal of the ESSKA. 2008;16(12):1121-32.
8. Bourne RB, Finlay JB, Papadopoulos P, Andreae P. The effect of medial meniscectomy on strain distribution in the proximal part of the tibia. J Bone Joint Surg Am. 1984;66(9):1431-7.
9. Ruiz-Iban MA, Diaz-Heredia J, Elias-Martin E, Moros-Marco S, Cebreiro Martinez Del Val I. Repair of meniscal tears associated with tibial plateau fractures: a review of 15 cases. The American journal of sports medicine. 2012;40(10):2289-95.
10. Poulsen MR, Johnson DL. Meniscal injuries in the young, athletically active patient. Phys Sportsmed. 2011;39(1):123-30.
11. Englund M, Guermazi A, Roemer FW, Yang M, Zhang Y, Nevitt MC, et al. Meniscal pathology on MRI increases the risk for both incident and enlarging subchondral bone marrow lesions of the knee: the MOST Study. Annals of the rheumatic diseases. 2010;69(10):1796-802.
12. Gauffin H, Tagesson S, Meunier A, Magnusson H, Kvist J. Knee arthroscopic surgery is beneficial to middle-aged patients with meniscal symptoms: a prospective, randomised, single-blinded study. Osteoarthritis Cartilage. 2014;22(11):1808-16.
13. Moseley JB, O'Malley K, Petersen NJ, Menke TJ, Brody BA, Kuykendall DH, et al. A controlled trial of arthroscopic surgery for osteoarthritis of the knee. N Engl J Med. 2002;347(2):81-8.
14. Abram SGF, Judge A, Beard DJ, Wilson HA, Price AJ. Temporal trends and regional variation in the rate of arthroscopic knee surgery in England: analysis of over 1.7 million procedures between 1997 and 2017. Has practice changed in response to new evidence? British journal of sports medicine. 2018.
15. Abrams GD, Frank RM, Gupta AK, Harris JD, McCormick FM, Cole BJ. Trends in meniscus repair and meniscectomy in the United States, 2005-2011. Am J Sports Med. 2013;41(10):2333-9.
16. Thorlund JB, Hare KB, Lohmander LS. Large increase in arthroscopic meniscus surgery in the middle-aged and older population in Denmark from 2000 to 2011. Acta Orthop. 2014;85(3):287-92.
17. Abram SGF, Beard DJ, Price AJ. National consensus on the definition, investigation, and classification of meniscal lesions of the knee. The Knee. 2018;25(5):834-40.
18. Thorlund JB, Juhl CB, Roos EM, Lohmander LS. Arthroscopic surgery for degenerative knee: systematic review and meta-analysis of benefits and harms. Br J Sports Med. 2015;49(19):1229-35.
19. Peterfy CG, Guermazi A, Zaim S, Tirman PF, Miaux Y, White D, et al. Whole-Organ Magnetic Resonance Imaging Score (WORMS) of the knee in osteoarthritis. Osteoarthritis and cartilage. 2004;12(3):177-90.
20. Bowes MA, Vincent GR, Wolstenholme CB, Conaghan PG. A novel method for bone area measurement provides new insights into osteoarthritis and its progression. Annals of the rheumatic diseases. 2015;74(3):519-25.
21. Kirkley A, Griffin S Fau - Whelan D, Whelan D. The development and validation of a quality of life-measurement tool for patients with meniscal pathology: the Western Ontario Meniscal Evaluation Tool (WOMET). (1050-642X (Print)).

For peer review only

22. Abram SA-O, Middleton R, Beard DJ, Price AJ, Hopewell S. Patient-reported outcome measures for patients with meniscal tears: a systematic review of measurement properties and evaluation with the COSMIN checklist. (2044-6055 (Electronic)).

23. Roos EM, Lohmander LS. The Knee injury and Osteoarthritis Outcome Score (KOOS): from joint injury to osteoarthritis. (1477-7525 (Electronic)).

24. Thorlund JB, Englund M, Christensen R, Nissen N, Pihl K, Jorgensen U, et al. Patient reported outcomes in patients undergoing arthroscopic partial meniscectomy for traumatic or degenerative meniscal tears: comparative prospective cohort study. BMJ. 2017;356:j356.

25. Gudbergsen H, Bartels Em Fau - Krusager P, Krusager P Fau - Waehrens EE, Waehrens Ee Fau - Christensen R, Christensen R Fau - Danneskiold-Samsoe B, Danneskiold-Samsoe B Fau - Bliddal H, et al. Test-retest of computerized health status questionnaires frequently used in the monitoring of knee osteoarthritis: a randomized crossover trial. (1471-2474 (Electronic)).

26. EuroQol. EQ-5D-5L Crosswalk index value calculator [Available from: https://euroqol.org/eq-5d-instruments/eq-5d-5l-about/valuation-standard-value-sets/crosswalk-index-value-calculator/.

27. EuroQol G. EuroQol--a new facility for the measurement of health-related quality of life. Health policy. 1990;16(3):199-208.

28. Brooks R. EuroQol: the current state of play. (0168-8510 (Print)).

29. Janssen MF, Pickard As Fau - Golicki D, Golicki D Fau - Gudex C, Gudex C Fau - Niewada M, Niewada M Fau - Scalone L, Scalone L Fau - Swinburn P, et al. Measurement properties of the EQ-5D-5L compared to the EQ-5D-3L across eight patient groups: a multi-country study. (1573-2649 (Electronic)).

30. Walters SJ, Brazier JE. Comparison of the minimally important difference for two health state utility measures: EQ-5D and SF-6D. Quality of life research : an international journal of quality of life aspects of treatment, care and rehabilitation. 2005;14(6):1523-32.

31. Kamper Sj Fau - Maher CG, Maher Cg Fau - Mackay G, Mackay G. Global rating of change scales: a review of strengths and weaknesses and considerations for design. (2042-6186 (Electronic)).

32. Barlow T, Dunbar M, Sprowson A, Parsons N, Griffin D. Development of an outcome prediction tool for patients considering a total knee replacement--the Knee Outcome Prediction Study (KOPS). BMC musculoskeletal disorders. 2014;15:451.

33. Champely S. pwr: Basic Functions for Power Analysis. R Package V1.2-2. https://cran.r-project.org/package=pwr; 2018.

34. Sedaghat AR. Understanding the Minimal Clinically Important Difference (MCID) of Patient-Reported Outcome Measures. Otolaryngol Head Neck Surg. 2019;161(4):551-60.

35. Team RC. A language and environment for statistical computing. R Foundation for Statistical Computing, Vienna, Austria. URL https://www.r-project.org/.

2018.

36. Staniszewska S, Brett J, Simera I, Seers K, Mockford C, Goodlad S, et al. GRIPP2 reporting checklists: tools to improve reporting of patient and public involvement in research. Research involvement and engagement. 2017;3:13.

For peer review only

Ymchwil Iechyd a Gofal Cymru
Health and Care Research Wales

NHS
Health Research Authority

Mr Andrew Metcalfe
Warwick Clinical Trials Unit
Clifford Bridge Road
Coventry
CV47AL

Email: hra.approval@nhs.net
Research-permissions@wales.nhs.uk

10 May 2019

Dear Mr. Metcalfe,

HRA and Health and Care Research Wales (HCRW) Approval Letter

**Study title:**	**The MEniscal TeaR Outcome (METRO) study: Understanding the disease process of young patients with a meniscal tear of the knee and response to treatment**
**IRAS project ID:**	**259098**
**Protocol number:**	**SC.08 18-19**
**REC reference:**	**19/WM/0079**
**Sponsor**	**University of Warwick**

I am pleased to confirm that **HRA and Health and Care Research Wales (HCRW) Approval** has been given for the above referenced study, on the basis described in the application form, protocol, supporting documentation and any clarifications received. You should not expect to receive anything further relating to this application.

Please now work with participating NHS organisations to confirm capacity and capability, in line with the instructions provided in the "Information to support study set up" section towards the end of this letter.

How should I work with participating NHS/HSC organisations in Northern Ireland and Scotland?

HRA and HCRW Approval does not apply to NHS/HSC organisations within Northern Ireland and Scotland.

If you indicated in your IRAS form that you do have participating organisations in either of these devolved administrations, the final document set and the study wide governance report (including this letter) have been sent to the coordinating centre of each participating nation. The relevant national coordinating function/s will contact you as appropriate.

For peer review only

Please see IRAS Help for information on working with NHS/HSC organisations in Northern Ireland and Scotland.

How should I work with participating non-NHS organisations?
HRA and HCRW Approval does not apply to non-NHS organisations. You should work with your non-NHS organisations to obtain local agreement in accordance with their procedures.

What are my notification responsibilities during the study?

The document "*After Ethical Review – guidance for sponsors and investigators*", issued with your REC favourable opinion, gives detailed guidance on reporting expectations for studies, including:

- Registration of research
- Notifying amendments
- Notifying the end of the study

The HRA website also provides guidance on these topics, and is updated in the light of changes in reporting expectations or procedures.

Who should I contact for further information?
Please do not hesitate to contact me for assistance with this application. My contact details are below.

Your IRAS project ID is **259098**. Please quote this on all correspondence.

Yours sincerely,
Laura Greenfield

Approvals Specialist

Email: hra.approval@nhs.net

Copy to: *Mrs Jane Prewett [Sponsor Contact]*

For peer review only

List of Documents

The final document set assessed and approved by HRA and HCRW Approval is listed below.

Document	Version	Date
Contract/Study Agreement template [mNCA]	2.1	12 July 2018
Covering letter on headed paper [METRO Cover letter]	2.0	20 March 2019
Evidence of Sponsor insurance or indemnity (non NHS Sponsors only) [University of Warwick combined TWIMC Cert]	1.0	27 September 2018
GP/consultant information sheets or letters [METRO Letter to GP]	1.0	07 February 2019
HRA Schedule of Events	2	05 March 2019
HRA Statement of Activities	1	05 March 2019
Interview schedules or topic guides for participants [METRO Interview Schedule V1.0]	1.0	07 February 2019
IRAS Application Form [IRAS_Form_21022019]		21 February 2019
IRAS Checklist XML [Checklist_21022019]		21 February 2019
Letter from funder [NIHR Letter of intent]	1.0	29 August 2018
Letter from sponsor [SC.08 18-19 Sponsorship Approval Letter]	1.0	11 February 2019
Participant consent form [METRO Consent form V1.0]	1.0	07 February 2019
Participant consent form [METRO Interview consent form V10]	1.0	07 February 2019
Participant information sheet (PIS) [METRO PIS V2.0]	2.0	20 March 2019
Participant information sheet (PIS) [METRO Interview PIS V2.0]	2.0	20 March 2019
Research protocol or project proposal [METRO protocol V1.0]	1.0	07 February 2019
Summary CV for Chief Investigator (CI) [Short CV Metcalfe]	1.0	07 February 2019
Summary CV for student [METRO IA 2 page CV]	1.0	07 February 2019
Summary CV for supervisor (student research) [Short CV Metcalfe]	1.0	07 February 2019
Summary of any applicable exclusions to sponsor insurance (non-NHS sponsors only) [University of Warwick Combined TWIMC Cert]	1.0	27 September 2018
Summary of any applicable exclusions to sponsor insurance (non-NHS sponsors only) [Warwick Insurance Letter]	1.0	27 July 2018
Summary, synopsis or diagram (flowchart) of protocol in non technical language [METRO Study flow chart]	1.0	07 February 2019
Validated questionnaire [METRO Baseline questionnaire V1.0]	1.0	07 February 2019
Validated questionnaire [METRO 3 month questionnaire V1.0]	1.0	07 February 2019
Validated questionnaire [METRO 6 month questionnaire V1.0]	1.0	07 February 2019
Validated questionnaire [METRO 12 month questionnaire V1.0]	1.0	07 February 2019

For peer review only

IRAS project ID	259098

Information to support study set up

The below provides all parties with information to support the arranging and confirming of capacity and capability with participating NHS organisations in England and Wales. This is intended to be an accurate reflection of the study at the time of issue of this letter.

Types of participating NHS organisation	Expectations related to confirmation of capacity and capability	Agreement to be used	Funding arrangements	Oversight expectations	HR Good Practice Resource Pack expectations
1 site type	Research activities should not commence at participating NHS organisations in England or Wales prior to their formal confirmation of capacity and capability to deliver the study.	A statement of activities has been submitted and the sponsor is intending to use a separate site agreement. The agreement has been modified as follows:<br>• Modification to clause 3.4 which is explained within the Statement of Activities Q5.<br>These changes are provided by the sponsor and the HRA and HCRW take no position on the acceptability of these changes. Participating NHS organisations should now determine its acceptability	Applicant has submitted a modified mNCA which contains the financial arrangements for the study.	A Principal Investigator is expected to be in place at the participating NHS site.	Should prior contractual arrangements with the host NHS sites not be in place, the researchers undertaking research activities at the NHS trusts would be expected to obtain Letters of Access on the basis of Research Passports if University employed, or NHS to NHS confirmation of preengagement checks letters if they are NHS employed, or have already Honorary Research Contracts. Standard DBS checks and occupational health clearance would be appropriate.

For peer review only

		and liaise with the sponsor to confirm the content of the agreement.			

Other information to aid study set-up and delivery

This details any other information that may be helpful to sponsors and participating NHS organisations in England and Wales in study set-up.
The applicant has indicated that they intend to apply for inclusion on the NIHR CRN Portfolio.

For peer review only

West Midlands - Black Country Research Ethics Committee

The Old Chapel
Royal Standard Place
Nottingham
NG1 6FS

Please note: This is the favourable opinion of the REC only and does not allow you to start your study at NHS sites in England until you receive HRA Approval

12 April 2019

Mr Andrew Metcalfe
Warwick Clinical Trials Unit
Clifford Bridge Road
Coventry
CV47AL

Dear Mr Metcalfe

**Study title:**	**The MEniscal TeaR Outcome (METRO) study: Understanding the disease process of young patients with a meniscal tear of the knee and response to treatment**
**REC reference:**	**19/WM/0079**
**Protocol number:**	**SC.08 18-19**
**IRAS project ID:**	**259098**

Thank you for your letter, responding to the Proportionate Review Sub-Committee's request for changes to the documentation for the above study.

The revised documentation has been reviewed and approved by the sub-committee.

We plan to publish your research summary wording for the above study on the HRA website, together with your contact details. Publication will be no earlier than three months from the date of this favourable opinion letter. The expectation is that this information will be published for all studies that receive an ethical opinion but should you wish to provide a substitute contact point, wish to make a request to defer, or require further information, please contact please contact hra.studyregistration@nhs.net outlining the reasons for your request.

For peer review only

Under very limited circumstances (e.g. for student research which has received an unfavourable opinion), it may be possible to grant an exemption to the publication of the study.

Confirmation of ethical opinion

On behalf of the Committee, I am pleased to confirm a favourable ethical opinion for the above research on the basis described in the application form, protocol and supporting documentation as revised.

Conditions of the favourable opinion

Management permission must be obtained from each host organisation prior to the start of the study at the site concerned.

Management permission should be sought from all NHS organisations involved in the study in accordance with NHS research governance arrangements. Each NHS organisation must confirm through the signing of agreements and/or other documents that it has given permission for the research to proceed (except where explicitly specified otherwise).

Guidance on applying for HRA and HCRW Approval (England and Wales)/ NHS permission for research is available in the Integrated Research Application System, at www.hra.nhs.uk or at http://www.rdforum.nhs.uk.

Where a NHS organisation's role in the study is limited to identifying and referring potential participants to research sites ("participant identification centre"), guidance should be sought from the R&D office on the information it requires to give permission for this activity.

For non-NHS sites, site management permission should be obtained in accordance with the procedures of the relevant host organisation.

Sponsors are not required to notify the Committee of management permissions from host organisations.

Registration of Clinical Trials

All clinical trials (defined as the first four categories on the IRAS filter page) must be registered on a publically accessible database. This should be before the first participant is recruited but no later than 6 weeks after recruitment of the first participant.

There is no requirement to separately notify the REC but you should do so at the earliest opportunity e.g. when submitting an amendment. We will audit the registration details as part of the annual progress reporting process.

To ensure transparency in research, we strongly recommend that all research is registered but for non-clinical trials this is not currently mandatory.

If a sponsor wishes to request a deferral for study registration within the required timeframe, they should contact hra.studyregistration@nhs.net. The expectation is that all clinical trials will be registered, however, in exceptional circumstances non registration may be permissible with prior agreement from the HRA. Guidance on where to register is provided on the HRA website.

It is the responsibility of the sponsor to ensure that all the conditions are complied with before the start of the study or its initiation at a particular site (as applicable).

For peer review only

Ethical review of research sites

The favourable opinion applies to all NHS sites taking part in the study, subject to management permission being obtained from the NHS/HSC R&D office prior to the start of the study (see "Conditions of the favourable opinion" above).

Approved documents

The documents reviewed and approved by the Committee are:

Document	*Version*	*Date*
Covering letter on headed paper [METRO Cover letter]	2.0	20 March 2019
Evidence of Sponsor insurance or indemnity (non NHS Sponsors only) [University of Warwick combined TWIMC Cert]	1.0	27 September 2018
GP/consultant information sheets or letters [METRO Letter to GP]	1.0	07 February 2019
Interview schedules or topic guides for participants [METRO Interview Schedule V1.0]	1.0	07 February 2019
IRAS Application Form [IRAS_Form_21022019]		21 February 2019
Letter from funder [NIHR Letter of intent]	1.0	29 August 2018
Letter from sponsor [SC.08 18-19 Sponsorship Approval Letter]	1.0	11 February 2019
Other [METRO response to provisional opinion V1.0]	1.0	20 March 2019
Participant consent form [METRO Consent form V1.0]	1.0	07 February 2019
Participant consent form [METRO Interview consent form V10]	1.0	07 February 2019
Participant information sheet (PIS) [METRO PIS V2.0]	2.0	20 March 2019
Participant information sheet (PIS) [METRO Interview PIS V2.0]	2.0	20 March 2019
Participant information sheet (PIS) [METRO PIS Interview]	2.0	20 March 2019
Participant information sheet (PIS) [METRO PIS]	2.0	20 March 2019
Referee's report or other scientific critique report [NIHR Feedback]	1.0	07 February 2019
Research protocol or project proposal [METRO protocol V1.0]	1.0	07 February 2019
Summary CV for Chief Investigator (CI) [Short CV Metcalfe]	1.0	07 February 2019
Summary CV for student [METRO IA 2 page CV]	1.0	07 February 2019
Summary CV for supervisor (student research) [Short CV Metcalfe]	1.0	07 February 2019
Summary of any applicable exclusions to sponsor insurance (non-NHS sponsors only) [University of Warwick Combined TWIMC Cert]	1.0	27 September 2018
Summary of any applicable exclusions to sponsor insurance (non-NHS sponsors only) [Warwick Insurance Letter]	1.0	27 July 2018
Summary, synopsis or diagram (flowchart) of protocol in non technical language [METRO Study flow chart]	1.0	07 February 2019
Validated questionnaire [METRO Baseline questionnaire V1.0]	1.0	07 February 2019
Validated questionnaire [METRO 3 month questionnaire V1.0]	1.0	07 February 2019
Validated questionnaire [METRO 6 month questionnaire V1.0]	1.0	07 February 2019
Validated questionnaire [METRO 12 month questionnaire V1.0]	1.0	07 February 2019

Statement of compliance

The Committee is constituted in accordance with the Governance Arrangements for Research Ethics Committees and complies fully with the Standard Operating Procedures for Research Ethics Committees in the UK.

For peer review only

After ethical review

Reporting requirements

The attached document "After ethical review – guidance for researchers" gives detailed guidance on reporting requirements for studies with a favourable opinion, including:

- Notifying substantial amendments
- Adding new sites and investigators
- Notification of serious breaches of the protocol
- Progress and safety reports
- Notifying the end of the study

The HRA website also provides guidance on these topics, which is updated in the light of changes in reporting requirements or procedures.

Feedback

You are invited to give your view of the service that you have received from the Research Ethics Service and the application procedure. If you wish to make your views known please use the feedback form available on the HRA website: http://www.hra.nhs.uk/about-the-hra/governance/quality-assurance

HRA Learning

We are pleased to welcome researchers and research staff to our HRA Learning Events and online learning opportunities– see details at: https://www.hra.nhs.uk/planning-and-improving-research/learning/

**19/WM/0079**	**Please quote this number on all correspondence**

With the Committee's best wishes for the success of this project.

Yours sincerely

Mr Nanak Singh Sarhadi
Chair

Email: nrescommittee.westmidlands-blackcountry@nhs.net

Enclosures: *"After ethical review – guidance for researchers"*

Copy to: *Mrs Jane Prewett*

Lead Nation England: HRA.Approval@nhs.net

For peer review only

National Institute for Health Research

NIHR Trainees Coordinating Centre
Leeds Innovation Centre
103 Clarendon Road
Leeds LS2 9DF

Tel: 0113 346 6260
www.nihr.ac.uk

Email: TCCawards@nihr.ac.uk

Mr Imran Ahmed
University of Warwick
Trauma and Orthopaedics
Clinical Science Research Laboratory
UHCW
Clifford Bridge Road
Coventry
CV22DX

29 August 2018

Dear Mr Ahmed,

NIHR Doctoral Research Fellowship - Stage 2
Award Ref: DRF-2018-11-ST2-030
Award Title: The MEniscal TeaR Outcome (METRO) Study: Understanding the disease process of young patients with a meniscal tear of the knee and response to treatment.

I am pleased to inform you that the NIHR Doctoral Research Fellowship - Stage 2 Panel has recommended your application for funding, and the Department of Health, in their capacity as the National Institute for Health Research (NIHR), has confirmed their intention to award funding. This is based upon acceptance of the terms and conditions set out in the Standard Research Contract (link below), and pending agreement to any conditions set by the Panel, details of which will have been communicated to you where relevant.

The Standard Research Contract, between Host Organisations and the Secretary of State for Health can be found on the NIHR website at:

https://www.nihr.ac.uk/funding-and-support/funding-for-training-and-career-development/training-programmes/useful-information-for-applicants/.

Next Steps

The NIHR is committed to the rapid initiation of research following the decision to fund, in order to ensure that the benefit to patients is realised as soon as possible. Therefore, we expect you and your host organisation to be working towards gaining the necessary contractual agreements and governance approvals required to start the award by 01 December 2018.

The NIHR acknowledges the risk to organisations around committing resource to research before a contract is in place; however, it is rare to not reach contractual terms unless the circumstances of the awardee change. The NIHR, therefore, encourages organisations to commit staff to setting up projects at as early an opportunity as possible, in order to expedite the formal commencement of research.

For peer review only

It is acknowledged that there can be unforeseen delays in starting up a research project, but in order to help reduce these it is your responsibility, with the support of your host organisation, to work closely with your organisation's R&D department or equivalent as well as other colleagues / departments involved in the administration and management of the research, and to start these discussions at the earliest opportunity.

To ensure that the award starts within the agreed timeframe, with all the required agreements and approvals in place, you as the awardee, and where necessary any other appropriate shared or support staff, need to be in post to enable the award to start on 01 December 2018. These staff costs will ultimately be covered through the research funding award, but your organisation is encouraged to meet them, where possible, from Research Capability Funding (RCF) prior to the research contract being agreed.

To support the often-iterative process towards agreement of the contract, we have set out the guiding timeframes for the submission of responses or information for each step towards the agreement of the Standard Research Contract as well as the anticipated start date.

- Confirmation of acceptance of funding – 2 weeks from the date of this letter
- Responses to Finance and IP queries – 2 weeks following the issue of queries
- Submission of draft collaboration agreements and/or subcontracts (where applicable) – 1 month prior to the contract start date
- Contract signature – 2 weeks prior to the contract start date
- Contracted start date – 01 December 2018

On receipt of information as set out above, the NIHR through the Trainees Coordinating Centre is committed to responding to your submission of information within two weeks or we will update you on progress.

Please take the time to carefully read the enclosures to this letter which detail the feedback on your application, your contact, Rebecca Savage, within the Trainees Coordinating Centre who will be working with you on the contract, the processes to be undertaken during the next steps, as well as additional information relating to your award.

Yours sincerely

Dr Lisa Cotterill
Director

Enclosures:

- Feedback – a summary of your reviews is provided separately.
- Trainee guidance which can be accessed here: https://www.nihr.ac.uk/funding-and-support/documents/Current%20Trainee-Guidance-PersonalAwardsJuly15-original.pdf
- Information for Themed Call applications. (Annex A).
- Additional information to support the contracting process. (Annex B).
- Ensuring publication of NIHR funded research (Annex C).
- CRN (Annex D).
- Your contact at NIHR (Annex E).
- Accepting your award offer (Annex F).

Cc: Dr Navdeep Bains

For peer review only

Annex A Themed Call applications

This information is only relevant to applicants who have been funded as part of an NIHR wide Themed Call.

If your proposal was funded as part of an NIHR Themed Call the Department of Health may wish to co-ordinate publicity about awards that fall within the scope of the themed call. As a result, the Department of Health has requested that projects funded directly from NIHR themed calls hold off from any individual project publicity until after this announcement has taken place in order to maximise the impact.

We understand that, in order for the new studies to begin recruiting, details will need to be placed in the public domain (e.g. on websites) and discussions held with organisations supporting or involved in the study. However, aside from activities relating to recruitment or project start up, we do ask that you do not actively publicise or promote the award. Please be assured that we will keep you informed of the status of the embargo and the dates of any publicity planned by the Department of Health.

FAQs

Q1: Can I issue a press release announcing my award?
R1: No – under the terms and conditions of the embargo you would not be able to issue a press release to announce that you had received funding from NIHR under a Themed Call.

Q2: Can I put up a poster in one of our recruitment sites (for example, a GP surgery) to let patients know we are recruiting to the trial?
R2: Yes – it is fine to issue publicity material to aid recruitment to the study as long as the focus of the release is to aid recruitment and not to solely announce the award. The embargo is not intended to jeopardize the success of the study in any way.

Q3: When will the embargo be lifted?
R3: We will let you know as soon as possible when the embargo is lifted by DH and then, as long as you have signed contracts, you will be free to issue a press release.

ew only

Annex B – Additional information to support the contracting process

The following information is provided to help speed up the process of setting up your award contract. By providing this information early it will reduce any delays in getting your contract agreed and signed.

Salary confirmation

As part of the contracting process NIHR TCC will re-calculate your salary to ensure it is accurate. In order for us to expedite this process we would be grateful if you could by return to TCCawards@nihr.ac.uk, provide the following information: 1. current salary, 2. increment date, 3. a copy of, or link to, your host organisation's salary scale. Please also provide any other salient information regarding your salary.

Contracts and Intellectual Property

A draft contract can be viewed through the link given above. It is recommended that the individual or department at your host organisation responsible for reading and signing off your contract looks at this draft contract at the earliest opportunity. This is to ensure they are aware of the terms and conditions of the funding and what the organisation will need to sign up to. We also strongly recommend the relevant people with intellectual property (IP) expertise at your host organisation look at schedules C and D of the draft contract which refer to the ownership of IP and agree the relevant wording for these sections. In our experience where third party or collaboration agreements need to be in place it can take a number of weeks to get this wording agreed so we suggest starting this process as early as possible. Please contact NIHR TCC if you have any queries. More information including some FAQs on intellectual property can be found on the NIHR website (https://www.nihr.ac.uk/funding-and-support/funding-for-research-studies/manage-my-study/contracts-and-intellectual-property.htm).

For peer review only

Annex C – Ensuring publication of NIHR-funded research

Current estimates suggest that 40-50% of research undertaken is never published.[1]

However, we expect that all NIHR funded research will be reported fully and publicly available when the research has been completed. The standard NIHR contract with researchers includes the following statement:
"17.7 The Contractor shall ensure that the outcome of the Research is prepared for publication in a suitable peer-reviewed journal"

We expect that all researchers who have a contract with the NIHR to undertake research shall ensure that the outcome of the research is prepared as a research paper for publication in a suitable peer-reviewed journal. There is also a contractual obligation that the researchers should, at the time of submission of their research paper to a peer-reviewed journal, send a copy of this to the NIHR Programme issuing the contract. This is to fulfil reporting requirements. It will also allow a mechanism by which NIHR Programmes can monitor the contractual obligation of researchers to prepare such a research paper on all NIHR funded research.

Full reporting should include: full title, structured abstract, full report to include aims, methods, results, conclusions and research recommendations. Depending on the publication, research materials should also be included, for example, patient questionnaires. Reporting must be in line with best guidance (eg CONSORT for clinical trials) according to study design listed on the EQUATOR website www.equator-network.org.

Publicly available means that the paper should be available on the web, able to be found using a recognised search facility, and as a minimum the abstract should be freely available.

It is expected that research funded by one of the NIHR Programmes that now has its own peer-reviewed journal as part of the NIHR Journals Library will publish a full and complete account of that research in the NIHR Programme specific journal. This will ensure that this research is reported fully, is publicly available with the abstract and full report freely available via the NIHR Journals Library website and the abstract freely available via Europe PubMed Central. The NIHR Programmes with journals in the NIHR Journals Library are: Efficacy and Mechanism Evaluation Programme; Health Technology Assessment Programme; Health Services and Delivery Research Programme; Public Health Research Programme; and Programme Grants for Applied Research.

We expect that research funded by a NIHR personal research training award shall ensure that the outcome of the research is prepared as a research paper for publication in a peer-reviewed journal. Where this research paper is not accepted for publication by a suitable peer-reviewed journal an alternative solution should be sought to ensure that this research is able to be reported fully and be publicly available.

We would also encourage all researchers to disseminate their research findings to the broader public community as well as to the research participants when the study has completed.

Reference:
1. Chalmers I, Glasziou P. Avoidable waste in the production and reporting of research evidence. Lancet 2009;374:86-89

For peer review only

Annex D - NIHR Clinical Research Network (CRN) Support

If your study involves the NHS or NHS patients, we expect you to apply, where appropriate, for NIHR CRN support and subsequent inclusion in the NIHR CRN Portfolio of studies to fully benefit from the support that the CRN offers through their Study Support Service. To find out more, please visit www.supportmystudy.nihr.ac.uk.

If your study is deemed eligible for consideration for NIHR Clinical Research Network support in England, and/or its equivalents in the Devolved Administrations, we expect you to:

- Keep your study record on the CRN's Central Portfolio management System (CPMS) up to date; and
- Upload your recruitment data into CPMS on a monthly basis. Please note that the NIHR Clinical Research Network will share this data with the NIHR TCC through the production of quarterly reports.

HRA Approval

If your study involves NHS sites in England you will need to apply for Health Research Authority Approval and follow the appropriate process for setting up sites. Please visit: http://www.hra.nhs.uk for further information.

For peer review only

Annex E – Your contact at NIHR

From the point of being awarded NIHR funding you will have an assigned Senior Programme Manager who will be your point of contact at NIHR for the duration of your award. Details of your assigned Senior Programme Manager are given below:

Senior Programme Manager: Rebecca Savage
Email address: rebecca.savage@nihr.ac.uk
Telephone: 0113 346 6260

For peer review only

Annex F – Accepting your award offer

It is important that your formally accept this offer of funding from NIHR and do so within 2 weeks of the date of this letter. If you are unable to respond within this time frame please contact NIHR via your named contact above at the earliest opportunity.

To accept this offer of funding please complete the online award acceptance form which can be accessed through your TCCi account (tcci.nihr.ac.uk). You will need to go to 'my reports' and select the 'Award Acceptance' report by clicking edit and follow the online instructions. The sooner this form is completed, the sooner we are able to start preparing a contract for your award.

For peer review only

BMJ Open

The Meniscal tear outcome study (METRO Study): A study protocol for a multicentre prospective cohort study exploring the factors which affect outcomes in patients with a meniscal tear.

Journal:	*BMJ Open*
Manuscript ID	bmjopen-2020-038681.R1
Article Type:	Protocol
Date Submitted by the Author:	01-Jun-2020
Complete List of Authors:	Ahmed, Imran; University of Warwick Warwick Medical School, Warwick Clinical trials Unit<br>Bowes, Mike; IMorphics Limited<br>Hutchinson, Charles; University of Warwick, Warwick Medical School<br>Parsons, Nicholas; University of Warwick, Warwick Medical School<br>Staniszewska, Sophie; University of Warwick, Warwick Medical School<br>Price, Andrew; University of Oxford, Nuffield Department of Orthopaedics, Rheumatology and Musculoskeletal Sciences<br>Metcalfe, Andrew; University of Warwick Warwick Medical School, Warwick Clinical Trials Unit; University Hospitals Coventry and Warwickshire NHS Trust, Trauma and Orthopaedics
**Primary Subject Heading**:	Surgery
Secondary Subject Heading:	Sports and exercise medicine
Keywords:	Adult orthopaedics < ORTHOPAEDIC & TRAUMA SURGERY, Knee < ORTHOPAEDIC & TRAUMA SURGERY, ORTHOPAEDIC & TRAUMA SURGERY

SCHOLARONE™
Manuscripts

I, the Submitting Author has the right to grant and does grant on behalf of all authors of the Work (as defined in the below author licence), an exclusive licence and/or a non-exclusive licence for contributions from authors who are: i) UK Crown employees; ii) where BMJ has agreed a CC-BY licence shall apply, and/or iii) in accordance with the terms applicable for US Federal Government officers or employees acting as part of their official duties; on a worldwide, perpetual, irrevocable, royalty-free basis to BMJ Publishing Group Ltd ("BMJ") its licensees and where the relevant Journal is co-owned by BMJ to the co-owners of the Journal, to publish the Work in this journal and any other BMJ products and to exploit all rights, as set out in our licence.

The Submitting Author accepts and understands that any supply made under these terms is made by BMJ to the Submitting Author unless you are acting as an employee on behalf of your employer or a postgraduate student of an affiliated institution which is paying any applicable article publishing charge ("APC") for Open Access articles. Where the Submitting Author wishes to make the Work available on an Open Access basis (and intends to pay the relevant APC), the terms of reuse of such Open Access shall be governed by a Creative Commons licence – details of these licences and which Creative Commons licence will apply to this Work are set out in our licence referred to above.

Other than as permitted in any relevant BMJ Author's Self Archiving Policies, I confirm this Work has not been accepted for publication elsewhere, is not being considered for publication elsewhere and does not duplicate material already published. I confirm all authors consent to publication of this Work and authorise the granting of this licence.

For peer review only

The Meniscal tear outcome study (METRO Study): A study protocol for a multicentre prospective cohort study exploring the factors which affect outcomes in patients with a meniscal tear.

Imran Ahmed (Imran.ahmed4@nhs.net), University of Warwick, Clinical Trials Unit, Coventry, CV22DX, UK

Mike Bowes (mike@imorphics.com), IMorphics, Manchester UK

Charles Hutchinson (c.e.hutchinson@warwick.ac.uk) University of Warwick, Warwick Medical School, Coventry, UK

Nicholas Parsons (nick.parsons@warwick.ac.uk) University of Warwick, Warwick Medical School, Coventry, CV4 7AL, UK

Sophie Staniszewska (sophie.staniszewska@warwick.ac.uk) University of Warwick, Warwick Medical School, Coventry, CV31 1DE, UK

Andrew Price (Andrew.price@ndorms.ox.ac.uk), University of Oxford, Nuffield department of Orthopaedics, rheumatology and musculoskeletal science, Botnar research centre, Nuffield Orthopaedic centre, Windmill Road, Headington Oxford OX3 7LD, UK

Andrew Metcalfe (a.metcalfe@warwick.ac.uk) University of Warwick, Warwick medical school, Warwick Clinical trials unit, Warwick medical school, Gibbet Hill road, Coventry, CV2 2DX, UK.

Corresponding author:

Imran Ahmed

NIHR Doctoral Research Fellow

University of Warwick

Warwick Medical School

Warwick Clinical Trials Unit

Coventry

CV2 2DX

Imran.ahmed4@nhs.net

Word Count: 4643

For peer review only

Abstract

Introduction

This study is designed to explore the baseline characteristics of patients under 55 years of age with a meniscal tear, and to describe the relationship between the baseline characteristics and patient reported outcome measures over 12 months. Research has highlighted the need for a trial to investigate the effectiveness of arthroscopic meniscectomy in younger patients. Before this trial, we need to understand the patient population in greater detail.

Methods and analysis

This is a multicentre prospective cohort study. Participants aged between 18 to 55 years with an MRI confirmed meniscal tear are eligible for inclusion. Baseline characteristics including age, body mass index, gender, participant-reported outcome measures (PROMs) duration of symptoms and magnetic resonance imaging (MRI) will be collected. The primary outcome measure is the Western Ontario Meniscal Evaluation Tool (WOMET) at 12 months. Secondary outcome measures will include PROMs such as EQ5D, Knee injury and osteoarthritis outcome score (KOOS) and patient global impression of change score at 3, 6 and 12 months.

Ethics and dissemination

The study obtained approval from the National Research Ethics Committee (NRES) West Midlands – Black Country research ethics committee (19/WM/0079) on 12th April 2019. The study is sponsored by the University of Warwick. The results will be disseminated via peer reviewed publication.

Registration details

UHCW R&D Reference: IA428119.

University of Warwick Sponsor ID: SC.08/18-19

Article summary

Strengths and limitations

- Prospective study design: The study is prospectively designed which will reduce the risk of recall bias amongst participants.
- Multicentre design: By recruiting participants from multiple centres across the country, we are obtaining data from a range of physiotherapists and surgeons. This will ensure that the findings are representative of current UK NHS practice.
- Clearly defined outcome measures: We have preselected our PROMS which are widely used in previous meniscal research. This will enable comparisons with previous studies and also pooling of results in future reviews.
- Comprehensive clinical and radiological assessment of participants: We are including several important baseline features. This includes clinical history directly related to current BASK treatment guidelines. We are also the first study to explore the relationship between WORMS score and PROMs in patients with a meniscal tear.

For peer review only

- Absence of objective clinical outcome measures: A limitation of this study is that we are not reporting clinical outcome measures e.g. range of motion in the knee, muscle strength and return to sport.

For peer review only

Introduction

Meniscal tears affect 60-70 per 100,000 of the population in the UK and account for 70,000 UK hospital admissions per year (1) (2). Over recent years, there has been a substantial increase in meniscus related literature, with a number studies questioning the effectiveness of surgical management(3-5).

The meniscus is a highly specialised c-shaped structure located within the knee (6). It has an important role in distributing load across the knee joint, preventing damage to the articular cartilage (7). The meniscus is susceptible to tears or damage. Meniscal tears typically result from high energy twisting injuries, such injuries often occur during sporting activities (8, 9). Meniscal pathology is also common in older people and is closely associated with osteoarthritis (OA) and age-related degeneration which can lead to changes in the histology of the menisci and their mechanical properties(10).

The current management of meniscal tears includes non-surgical options such as provision of advice and observation, physiotherapy or pharmacological options such as anti-inflammatory medication or intra-articular corticosteroid injections. Surgical options include meniscal repair (in limited cases) or arthroscopic partial meniscectomy (APM), a keyhole procedure performed with the intention of removing the torn or unstable meniscus which may be thought to cause pain or mechanical symptoms e.g. locking (11).

Previous randomised controlled trials have found no evidence for a difference in outcome between APM and physiotherapy in patients with a meniscal tear and co-existing arthritis (12, 13). This led to a change in NICE guidelines advising against arthroscopic surgery to treat meniscal tears in the context of established arthritis (14) . Abram et al (2019) performed a systematic review including studies comparing arthroscopic meniscectomy versus a comparator in patients with a meniscal tear(4). The authors included 10 studies for analysis and performed subgroup analysis for patients without OA. The authors reported a small improvement in knee pain for all patients with a meniscal tear in the meniscectomy group compared to the non-surgical group (standardised mean difference (SMD) 0.22 95% CI 0.04 to 0.4). Three studies reported pain scores in patients without OA, in which the authors found an increased improvement in pain scores in the meniscectomy group (SMD 0.35 95% CI 0.04 to 0.66). There was a similar improvement in knee function in the meniscectomy group in patients without OA compared to all patients undergoing meniscectomy (SMD 0.18 vs SMD 0.3).

Despite the evidence challenging the clinical effectiveness of APM, there has been a 22% increase in the number of APMs performed over the last 20 years, from 151/100,000 in 1997 to 184/100,000 in 2017 (15-17). Previous literature has provided evidence on the lack of effectiveness of APM in patients with a degenerative knee or arthritis as the symptoms may be caused by the degeneration and not the meniscal tear(18, 19). UK national consensus statement from British Association for Surgery of the Knee (BASK) has highlighted the importance of patient factors which are important in the management of meniscal tears. The guidelines state patients with a particular pattern of meniscal tear visible on MRI ('a meniscal target') and the corresponding pattern of symptoms or signs should be recommended for non-urgent arthroscopy. Furthermore, patients should undergo a period of non-operative management before referral for APM (20). These guidelines also suggest that radiology investigations should be included in treatment decisions, with certain patterns of

For peer review only

tears being ‘target’ lesions(20). A future cohort study will need to assess the importance of the MRI tear pattern as an outcome predictor.

A well-designed cohort study needs to be performed with patients being recruited at the point of diagnosis or presentation to an orthopaedic specialist. This will allow researchers to include patients being managed non-operatively. By including MRI analysis in a potential predictive model, this will produce a comprehensive model to identify which features can predict variability in PROMs.

Study Aim

To describe the baseline characteristics and imaging findings of young patients with a meniscal tear and to explore the relationship, if any, between these baseline features and 12-month outcomes.

We will address the following research objectives:

1. Describe the baseline characteristics, pattern of symptoms and imaging findings of younger people (aged < 55 years) with a meniscal tear (study population)

2. Explore the relationship between these features and 12-month PROMS.

Methods and Analysis

A multi-centre prospective cohort study will be performed. The study obtained approval from the National Research Ethics Committee (NRES) West Midlands – Black Country research ethics committee (19/WM/0079) on 12th April 2019. All patients presenting to a secondary care centre (elective knee clinic or acute knee clinic) with a magnetic resonance imaging (MRI) confirmed meniscal tear, under the care of orthopaedic consultants at any of the participating sites, will be invited to take part in the study. The following eligibility will be implemented for patient selection.

Inclusion criteria:

- Age between 18 and 55 years
- Presence of an MRI confirmed meniscal tear
- Provision of informed written consent

Exclusion criteria:

- Anterior cruciate ligament or other major knee ligament injury. This does not include a previous unrelated healed medial collateral ligament (MCL) tear or a meniscal root tear (which is considered a type of meniscal tear in this study)
- Associated intra-articular fracture of the tibial plateau or femur. Previous fractures not thought to be related to the tear are not an exclusion criteria for the study
- Previous knee surgery, e.g unicondylar knee replacement; total knee replacement; knee arthroscopy; meniscal repair or meniscectomy.
- Previous entry into the present study (i.e. the other knee)
- Participant is unable to undertake study procedures

Participant identification and screening

For peer review only

Potential participants will be identified by a member of the attending clinical team or a member of the clinical research team by screening clinic lists in secondary or intermediate care clinics. This will involve a member of the clinical team reviewing letters for the upcoming knee or orthopaedic clinics to identify potential participants.

Eligibility will be confirmed based on assessment and standard care MRI. In normal clinical practice, MRI is a requirement for diagnosis of a meniscal tear, therefore all participants entering the study will already have had one at the time of entry into the study.

All individuals who meet the study entry criteria will be checked for eligibility and recorded on the monthly screening log. Eligible potential participants who are willing to be approached by a suitably trained member of the research team will be provided with verbal and written information about the study, and will have the opportunity to discuss and ask questions.

Baseline data collection

Once participants have provided informed consent, baseline data will be collected by the research team. Baseline characteristics such as age, gender, body mass index and date of potential injury and duration of symptoms will be collected. Baseline PROMs will be collected at recruitment in order to assess changes in PROMs at 12 months. Clinical features which are consistent with a treatable meniscal lesion, outlined by the BASK consensus meeting, will also be collected from the attending clinician (for a locked knee) and the participant (for locking and catching (20). These include the presence of:

- Locked knee: sudden onset, complete mechanical block to flexion or extension of the knee, detected on clinical examination and which does not resolve despite adequate analgesia.
- Locking: an intermittent block to normal range of movement of the knee (commonly a block to extension) with an associated unlocking movement. Knee returns to near normal after unlocking.
- Catching: the sensation of something intermittently out of place in the knee and interfering with joint movement.

These will be assessed on initial baseline questionnaires which will be provided by the host site. If the clinician documents specific examination findings (in particular alignment), this will be collected by the study team.

MRI scans will be analysed to assess:

1. Anatomical location and tear pattern. Tear patterns include the following which have been recognised by BASK as potentially treatable 'meniscal targets'(20).

   a) Bucket handle tear: a longitudinal tear involving more than 25% of the meniscus length (can be displaced or undisplaced)
   b) Displaced meniscal tear: fragments are displaced from their usual anatomical position
   c) Meniscal root tear
   d) Radial tear: a vertical tear which may or may not extend into the meniscocapsular junction
   e) Horizontal tear
   f) Complex tear: a meniscal lesion with more than one place of tear in continuity

For peer review only

2. Whole-Organ Magnetic Resonance Imaging Score (WORMS): This is a semi-quantitative, multi-feature scoring method for whole-organ evaluation of the knee using conventional MRI. It is based on 14 features include cartilage and meniscal integrity (21).

3. Bone area: analysis of bone area and cartilage volume using proprietary semi-automated segmentation software in collaboration with IMorphics (IMorphics Limited, Stryker House, Hambridge road, Newbury, Berkshire). Previous research has demonstrated that change in bone area is a more sensitive marker of osteoarthritis than cartilage thickness or joint space narrowing. This change in bone area could lead to the development of meniscal tears as flattening of the femoral condyls could potentially reduce the space available for the meniscus increasing the likelihood of meniscal damage (22).

Each MRI will be reported by an orthopaedic surgical trainee (IA) trained to report WORMS scores and a consultant radiologist (CEH).

Primary outcome:

Western Ontario Meniscal Evaluation Tool (WOMET) at 12 months. This is a meniscal tear disease-specific quality of life measure developed in collaboration with patients in 2007. It consists of 16 questions (items) focussing on three domains: (i) physical symptoms, (ii) sport/recreation/work/lifestyle and (iii) emotions. Items are assessed on a visual analogue scale (VAS), summed and reported as a percentage of the maximum score of 1600 (23). A recent systematic review of PROMs used for meniscal research demonstrated that WOMET had the strongest evidence for content validity (24). It is also one of the only PROMs developed with the involvement of patients with meniscal tears as their primary diagnosis.

Secondary outcomes

- **WOMET:** collected at baseline, 3 and 6 months: as outlined above.
- **Knee injury and Osteoarthritis outcome score 4 (KOOS)(26):** The KOOS questionnaire was developed in 1998 as a knee injury specific outcome measure of patients at risk of developing arthritis(25). Domains are pain, symptoms, functional status, sports activity and quality of life. It is used extensively in knee injury research, and has been the primary outcome in the majority of clinical trials studying the treatment of meniscal tears. In previous meniscal tear research a shorter form of KOOS ($KOOS_4$) has been widely used (26). This focuses on four out of five of the domains pain, symptoms, sport and recreational function and quality of life. Standardised answer options are provided using a Likert scale and each question is assigned a score of 0 – 4. A score of 100 indicates no symptoms whereas a score of 0 indicates extreme symptoms. This outcome tool can be used as a postal or electronic survey and both versions are comparable with regards to psychometrics (27). KOOS4 will be collected at baseline and 12 months only.
- **EuroQol (EQ-5D-5L):** This is a validated measure of health-related quality of life, consisting of a five-dimension health status classification system and a separate visual analogue scale. EQ-5D is applicable to a wide range of health conditions and treatments and provides a simple descriptive profile and a single index value for health status, range from -0.594 to 1, and anchored at zero (death) (28, 29). EQ-5D is primarily designed for self-completion by respondents and is ideally suited for use in postal surveys, in clinics and face-to-face

For peer review only

interviews (30, 31). It is cognitively simple, taking only a few minutes to complete (29, 32). This will be collected at baseline, 3 months, 6 months and 12 months.

- **Patient global assessment of change (PGIC):** A simple 7-point scale assessing participant perception of improvement (33). A standardised question will be used to explore the change in activity limitations, symptoms and quality of life in the painful knee. Responses will range from 'no change or condition has worsened' to 'a great deal better. This will provide valuable information for a future large scale trial by providing information on the minimally clinical important difference in WOMET score for this patient population. This will be collected at 3 months, 6 months and 12 months.
- **Surgery:** We will also collect data on whether participants undergo surgery for their meniscal tear. At each time point (3, 6 and 12 months) patients will be asked if they underwent surgery. We will continue to follow up patients after surgery to determine their outcome in a pragmatic fashion, as would happen in the conservative arm of a large trial.

These PROMs will be collected at baseline, 3, 6 and 12 months from the date of recruitment. Data will be collected using questionnaires designed with patient and public involvement. This data will be collected by a number of methods (either postal, telephone, electronically or face to face) dependent on participant preference. If a participant requests face-to-face consultation or has difficulty reading or writing, a clinic visit will be arranged.

Sample size calculation

Previous experience (34) for studies of this type, where statistical models have been built to explain variation in PROMs, suggests that they rarely explain more than 10-20% of the variation in outcomes (R^2) at 12 months. Using a large (and therefore rather conservative) estimate for the numerator number of degrees of freedom of 25 (i.e. the model complexity) for a putative F test of the model significance and an $R^2 = 0.1$, suggests a sample size of 160 for 90% power at the 5% significance level (https://cran.r-project.org/web/packages/pwr/; pwr.f2.test) (35). Experience of previous such studies in similar settings have reported follow up rates of 80%. Assuming loss to follow up of 20%, 200 participants will be required for this study.

Statistical analysis

Summary statistics will be used to describe baseline data. We will report means and standard deviations (95% confidence intervals) for approximately normally distributed data and for other cases report the median and interquartile range. Mean 12-month PROMs scores will be reported for the whole cohort. We will also report mean 12 month PROMs for both the non-operative and operative group. Pearson correlation coefficients will be used to assess evidence for associations between baseline features and 12 month PROMs scores. A model will be developed relating the response variable (12-month PROM) to a number of explanatory variables (e.g. data collected at baseline). The type and structure of the models used will depend on the distribution of the response variable, and the nature of the association between the response and explanatory variable. A Linear model will be the initial choice in order to predict the response variable (12-month PROM) as a linear

For peer review only

weighted sum of the explanatory variables. The ‘weights’ or parameters of this model are estimated using regression analysis; providing us with regression coefficients (parameter estimates), that characterise the association.

A sensitivity and specificity-based longitudinal anchor method will be used to calculate the MCID. The change in WOMET scores will be calculated for each group on the PGIC scale. This will be done by subtracting the 12 month WOMET from the baseline score. A mean change in WOMET score will be associated with each anchor on the PGIC as follows:

1. No change (or condition has got worse)
2. Almost the same, hardly any change at all
3. A little better, but no noticeable change
4. Somewhat better, but the change has not made any real difference
5. Moderately better, and slight but noticeable change
6. Better, and a definite improvement that has made a real and worthwhile difference
7. A great deal better, and a considerable improvement that has made all the difference

We will then use the receiver operator characteristics (ROC) to determine the MCID with equal sensitivity and specificity to discriminate between the changed group (5-7) and the unchanged group (1-4). The area under the ROC curve represents the probability the score discriminates between the unchanged and the changed group. A probability of >0.7 was deemed acceptable.

This methodology has been previously used in orthopaedic research and has been described in previous literature (36, 37).

Model fitting and analysis will be undertaken in R (R Core team (2013) R Foundation for statistical computing, Vienna, Austria) (38).

Discussion

This prospective cohort study aims to describe the baseline characteristics of young people (age<55) with a meniscal tear and to explore the relationship between these features at outcome. This study has a role in informing treatment decisions by identifying in certain patient features can predict treatment outcome. There is a view amongst researchers and clinicians that surgery may be beneficial amongst a particular subset of patients and further research is needed to identify this subset (4). This work will assist in the future planning of a randomised controlled trial by further exploring the study population. The study aims to identify the patients that improve with both operative and non-operative management and whether there are certain factors which influence the response to either treatment. It may be that in a population of patients who do not improve with non-operative care and do not have features of arthritis, that a study is required to assess the effectiveness of surgery, but before this can be performed, we need a much better understanding of the characteristics of this population and how they might be defined. This cohort study will also inform a future trial by providing an insight into follow-up rates in this younger population, efficient means of collecting outcome data, and also providing an understanding of the MCID of the WOMET score.

This study aims to build on a previous cohort study which was performed in order to identify features which may predict variability in patient reported outcomes (PROMs) for patients following arthroscopy. The authors included 18 preoperative factors and found the strongest predictive

For peer review only

factors were no previous meniscal surgery and more severe preoperative knee symptoms. However, factors such as tear pattern or structural osteoarthritis were not assessed as pre-operative imaging was not available to the authors, and so our study will answer a related, but different question. The models overall predictive performance was very low (optimum adjusted R^2=0.080) suggesting the included factors had little relevance to outcome(5). The authors only included patients undergoing operative intervention, work is needed to identify outcomes in all patients that are managed with a meniscal tear, in order to identify which patients performed well with non-operative management and which perform poorly.

An upper age limit of 55 was selected, as the mean age in a recent meta-analysis of trials in meniscal tears. As the one of the purposes of the study is to explore the relationship between baseline data and outcome, we will explore the effects on age. Age was not an important factor in the recently published analysis of the KACS cohort and whilst it may be relevant, it may also be a poor proxy for other relevant factors, such as underlying osteoarthritis or meniscal degeneration. This will be explored in this study and, if age is important, the study will allow us to set evidence-based thresholds for future research.

Strengths of the METRO cohort include its prospective nature, minimising the risk of participant recall bias. We are also reporting outcomes in all patients with a meniscal tear including both the operative and the non-operative group, this ensures the study results are more representative of all patients with a meniscal tear. The multi-centre national nature of this study ensures that the data included is generalizable to current UK practice, especially after publication of the national treatment guidelines (20). Further strengths include the use of MRI based assessment of tear pattern, arthritis and bone shape change which could be an important predictive factor for outcome.

Limitations of this study design in comparison to previous research is our sample size is smaller than a previous study which did not identify any predictive factors (5), however, this study did not include MRI data and we have included a thorough sample size calculation with a conservative estimate of the number of degrees of freedom. In addition, this observational study will provide an insight into associations between patient factors and PROMs, however as the study is observational the findings will be utilised for generation of new hypotheses and causal inference will be limited, and will require further study in randomised controlled trials. A multicentre RCT is currently recruitment comparing surgery versus physiotherapy, however, the eligibility criteria does not take into account presence of osteoarthritis on MRI(39). This cohort study will explore whether that is an important factor in patient outcome.

There is a need for further RCTs in a specific subset of patients where surgery may be of some benefit. However, we believe this study is in important, not only in informing current treatment pathways and adding further evidence to national guidelines but also contributing considerably to the planning of a future randomised trial assessing the effectiveness of arthroscopic meniscectomy in this young population.

Patient and public involvement

Eighteen patients with knee pain or osteoarthritis were consulted prior to study set up. Twelve of these patients had a meniscal tear. The PPI group provided valuable input on study set up such as

For peer review only

sources of recruitment, completion of case report forms, completion of questionnaires and how to conduct follow up. These patients also reviewed the language used in patient information sheets and consent forms to ensure it was appropriate for the target population.

We are in the process of setting up a PPI reference group of five patients with a meniscal tear. They will help address any issues which arise during the study.

Patient and public members will be involved in the interpretation of the results and facilitate the dissemination of the study results by helping to prepare patient leaflets, manuscripts for peer reviewed publication and dissemination through social media.

Patient and public members will also be regularly updated on study progress and will provide advice on issues including recruitment.

PPI will be reported using GRIPP2 reporting checklist(40).

Ethics and dissemination

The study obtained approval from the National Research Ethics Committee (NRES) West Midlands – Black Country research ethics committee (19/WM/0079) on 12th April 2019.

We will aim to publish the results in a peer reviewed journal, within 12 months of the study completing (Recruitment began in April 2019 and will continue to November 2020). The results of the study will also be disseminated via patient information material produced in collaboration with the PPI group. All key study findings will be presented at national and international conferences, e.g. British Orthopaedic Association (BOA) or British Association of Specialist Knee surgeons (BASK), and the American Academy of Orthopaedic Surgeons (AAOS).

Acknowledgements

We would like to acknowledge the support of:

1. The University of Warwick (Lead Sponsor)
2. University Hospital Coventry and Warwickshire (Lead NHS site).
3. Members of the PPI group.
4. Research and Clinical staff at all recruiting site: University Hospital Coventry and Warwickshire, George Elliot Hospital NHS Trust, University Hospitals Birmingham NHS Trust, Royal Orthoapedic Hospital, Birmingham Community Healthcare NHS trust, Robert Jones and Agnes Hunt NHS trust, Oxford University Hospitals, Imperial College NHS trust and North Bristol NHS trust.

Author contributions

IA: Study conception, study design, drafted and reviewed final manuscript.

MB: Study design, drafted and approved final manuscript.

CH: Study design, drafted and approved final manuscript.

NP: Study design, drafted and approved final manuscript.

SS: Study design, drafted and approved final manuscript.

For peer review only

AP: Study design, drafted and approved final manuscript.

AM: Chief Investigator, Study design, drafted and approved final manuscript.

Funding statement

The study protocol represents research funded by a National Institute for Health Research (NIHR) Doctoral Fellowship Award (DRF-2018-11-ST2-030). The study is sponsored by the University of Warwick. The study sponsor provides ultimate approval of all new versions of the protocol before they become live. Both the funder and sponsor are required to provide final approval before publication of any study material.

Data management and sharing

All data collected will be anonymised after the collection of the baseline demographic data for each participant. Confidentiality will be strictly maintained and names or addresses will not be disclosed to anyone other than the staff involved in running the study. Once participants are recruited into the study they will be registered onto a secure electronic database created by Warwick CTU. A trial number (TNO) will be provided for each participant. For all future follow questionnaires participants will be identified by TNO and initials. Access to the database will only be provided to METRO specific research staff at each NHS staff. At the CTU, the study lead (IA), chief investigator (AM) and statistician (NP) will have access to the electronic database. Identifiable participant data will be held in a locked filing cabinet and coded with the study number to tag identifiable data to the outcome data, only IA and AM will have access to this locked filing cabinet.

Direct access to source data/documents will be available for study-related monitoring or audit by Warwick CTU for internal audit, regulatory authorities or ethics committees.

Data will be available for non-commercial companies upon reasonable request.

Disclaimer

The study funder and sponsor had no role in the study design; the collection, analysis, or interpretation of data; the writing of the report; or the decision to submit for publication. The researchers are independent and the views expressed are those of the authors and not necessarily those of the NHS, the NIHR or the Department of Health.

Competing interests statement

Mike Bowes is a senior director of clinical applications at IMorphics limited.

References

1. Logerstedt DS, Snyder-Mackler L, Ritter RC, Axe MJ. Knee pain and mobility impairments: meniscal and articular cartilage lesions. The Journal of orthopaedic and sports physical therapy. 2010;40(6):A1-a35.
2. Abram SGF, Judge A, Beard DJ, Price AJ. Adverse outcomes after arthroscopic partial meniscectomy: a study of 700 000 procedures in the national Hospital Episode Statistics database for England. Lancet. 2018;392(10160):2194-202.
3. Abram SGF, Beard DJ, Price AJ. Arthroscopic meniscal surgery: a national society treatment guideline and consensus statement. Bone Joint J. 2019;101-B(6):652-9.

For peer review only

4. Abram SGF, Hopewell S, Monk AP, Bayliss LE, Beard DJ, Price AJ. Arthroscopic partial meniscectomy for meniscal tears of the knee: a systematic review and meta-analysis. Br J Sports Med. 2019.
5. Pihl K, Ensor J, Peat G, Englund M, Lohmander S, Jorgensen U, et al. Wild goose chase - no predictable patient subgroups benefit from meniscal surgery: patient-reported outcomes of 641 patients 1 year after surgery. Br J Sports Med. 2019.
6. Smith NA, Costa ML, Spalding T. Meniscal allograft transplantation: rationale for treatment. The bone & joint journal. 2015;97-b(5):590-4.
7. Masouros SD, McDermott ID, Amis AA, Bull AM. Biomechanics of the meniscus-meniscal ligament construct of the knee. Knee surgery, sports traumatology, arthroscopy : official journal of the ESSKA. 2008;16(12):1121-32.
8. Ruiz-Iban MA, Diaz-Heredia J, Elias-Martin E, Moros-Marco S, Cebreiro Martinez Del Val I. Repair of meniscal tears associated with tibial plateau fractures: a review of 15 cases. The American journal of sports medicine. 2012;40(10):2289-95.
9. Poulsen MR, Johnson DL. Meniscal injuries in the young, athletically active patient. Phys Sportsmed. 2011;39(1):123-30.
10. Englund M, Guermazi A, Roemer FW, Yang M, Zhang Y, Nevitt MC, et al. Meniscal pathology on MRI increases the risk for both incident and enlarging subchondral bone marrow lesions of the knee: the MOST Study. Annals of the rheumatic diseases. 2010;69(10):1796-802.
11. Gauffin H, Tagesson S, Meunier A, Magnusson H, Kvist J. Knee arthroscopic surgery is beneficial to middle-aged patients with meniscal symptoms: a prospective, randomised, single-blinded study. Osteoarthritis Cartilage. 2014;22(11):1808-16.
12. Moseley JB, O'Malley K, Petersen NJ, Menke TJ, Brody BA, Kuykendall DH, et al. A controlled trial of arthroscopic surgery for osteoarthritis of the knee. N Engl J Med. 2002;347(2):81-8.
13. Kirkley A, Birmingham TB, Litchfield RB, Giffin JR, Willits KR, Wong CJ, et al. A randomized trial of arthroscopic surgery for osteoarthritis of the knee. N Engl J Med. 2008;359(11):1097-107.
14. NICE. Osteoarthritis care and management cg177 2014 [Available from: https://www.nice.org.uk/guidance/cg177/chapter/1-recommendations.
15. Abram SGF, Judge A, Beard DJ, Wilson HA, Price AJ. Temporal trends and regional variation in the rate of arthroscopic knee surgery in England: analysis of over 1.7 million procedures between 1997 and 2017. Has practice changed in response to new evidence? British journal of sports medicine. 2018.
16. Abrams GD, Frank RM, Gupta AK, Harris JD, McCormick FM, Cole BJ. Trends in meniscus repair and meniscectomy in the United States, 2005-2011. Am J Sports Med. 2013;41(10):2333-9.
17. Thorlund JB, Hare KB, Lohmander LS. Large increase in arthroscopic meniscus surgery in the middle-aged and older population in Denmark from 2000 to 2011. Acta Orthop. 2014;85(3):287-92.
18. Katz JNL, E. Arthroscopic partial meniscectomy for degenerative tears: Where do we stand? Osteoarthritis Cartilage. 2014;22(11):1749-51.
19. Englund M, Guermazi A, Gale D, Hunter DJ, Aliabadi P, Clancy M, et al. Incidental meniscal findings on knee MRI in middle-aged and elderly persons. N Engl J Med. 2008;359(11):1108-15.
20. Abram SGF, Beard DJ, Price AJ. National consensus on the definition, investigation, and classification of meniscal lesions of the knee. Knee. 2018;25(5):834-40.
21. Peterfy CG, Guermazi A, Zaim S, Tirman PF, Miaux Y, White D, et al. Whole-Organ Magnetic Resonance Imaging Score (WORMS) of the knee in osteoarthritis. Osteoarthritis and cartilage. 2004;12(3):177-90.
22. Bowes MA, Vincent GR, Wolstenholme CB, Conaghan PG. A novel method for bone area measurement provides new insights into osteoarthritis and its progression. Annals of the rheumatic diseases. 2015;74(3):519-25.
23. Kirkley A, Griffin S Fau - Whelan D, Whelan D. The development and validation of a quality of life-measurement tool for patients with meniscal pathology: the Western Ontario Meniscal Evaluation Tool (WOMET). (1050-642X (Print)).

For peer review only

24. Abram SA-O, Middleton R, Beard DJ, Price AJ, Hopewell S. Patient-reported outcome measures for patients with meniscal tears: a systematic review of measurement properties and evaluation with the COSMIN checklist. (2044-6055 (Electronic)).
25. Roos EM, Lohmander LS. The Knee injury and Osteoarthritis Outcome Score (KOOS): from joint injury to osteoarthritis. (1477-7525 (Electronic)).
26. Thorlund JB, Englund M, Christensen R, Nissen N, Pihl K, Jorgensen U, et al. Patient reported outcomes in patients undergoing arthroscopic partial meniscectomy for traumatic or degenerative meniscal tears: comparative prospective cohort study. BMJ. 2017;356:j356.
27. Gudbergsen H, Bartels Em Fau - Krusager P, Krusager P Fau - Waehrens EE, Waehrens Ee Fau - Christensen R, Christensen R Fau - Danneskiold-Samsoe B, Danneskiold-Samsoe B Fau - Bliddal H, et al. Test-retest of computerized health status questionnaires frequently used in the monitoring of knee osteoarthritis: a randomized crossover trial. (1471-2474 (Electronic)).
28. EuroQol. EQ-5D-5L Crosswalk index value calculator [Available from: https://euroqol.org/eq-5d-instruments/eq-5d-5l-about/valuation-standard-value-sets/crosswalk-index-value-calculator/.
29. EuroQol G. EuroQol--a new facility for the measurement of health-related quality of life. Health Policy. 1990;16(3):199-208.
30. Brooks R. EuroQol: the current state of play. (0168-8510 (Print)).
31. Janssen MF, Pickard As Fau - Golicki D, Golicki D Fau - Gudex C, Gudex C Fau - Niewada M, Niewada M Fau - Scalone L, Scalone L Fau - Swinburn P, et al. Measurement properties of the EQ-5D-5L compared to the EQ-5D-3L across eight patient groups: a multi-country study. (1573-2649 (Electronic)).
32. Walters SJ, Brazier JE. Comparison of the minimally important difference for two health state utility measures: EQ-5D and SF-6D. Quality of life research : an international journal of quality of life aspects of treatment, care and rehabilitation. 2005;14(6):1523-32.
33. Kamper Sj Fau - Maher CG, Maher Cg Fau - Mackay G, Mackay G. Global rating of change scales: a review of strengths and weaknesses and considerations for design. (2042-6186 (Electronic)).
34. Barlow T, Dunbar M, Sprowson A, Parsons N, Griffin D. Development of an outcome prediction tool for patients considering a total knee replacement--the Knee Outcome Prediction Study (KOPS). BMC musculoskeletal disorders. 2014;15:451.
35. Champely S. pwr: Basic Functions for Power Analysis. R Package V1.2-2. https://cran.r-project.org/package=pwr; 2018.
36. Sedaghat AR. Understanding the Minimal Clinically Important Difference (MCID) of Patient-Reported Outcome Measures. Otolaryngol Head Neck Surg. 2019;161(4):551-60.
37. Copay AG, Subach BR, Glassman SD, Polly DW, Jr., Schuler TC. Understanding the minimum clinically important difference: a review of concepts and methods. The spine journal : official journal of the North American Spine Society. 2007;7(5):541-6.
38. Team RC. A language and environment for statistical computing. R Foundation for Statistical Computing, Vienna, Austria. URL https://www.r-project.org/.

2018.
39. Skou ST, Lind M, Hölmich P, Jensen HP, Jensen C, Afzal M, et al. Study protocol for a randomised controlled trial of meniscal surgery compared with exercise and patient education for treatment of meniscal tears in young adults. BMJ Open. 2017;7(8):e017436.
40. Staniszewska S, Brett J, Simera I, Seers K, Mockford C, Goodlad S, et al. GRIPP2 reporting checklists: tools to improve reporting of patient and public involvement in research. Research involvement and engagement. 2017;3:13.

For peer review only
